# A Unique Box in 28S rRNA Is Shared by the Enigmatic Insect Order Zoraptera and Dictyoptera

**DOI:** 10.1371/journal.pone.0053679

**Published:** 2013-01-03

**Authors:** Yanhui Wang, Michael S. Engel, Jose A. Rafael, Kai Dang, Haoyang Wu, Ying Wang, Qiang Xie, Wenjun Bu

**Affiliations:** 1 Institute of Entomology, College of Life Sciences, Nankai University, Tianjin, China; 2 Division of Entomology (Paleoentomology), Natural History Museum, London, England; 3 Department of Ecology and Evolutionary Biology, University of Kansas, Lawrence, Kansas, United States of America; 4 Instituto Nacional de Pesquisas da Amazônia, INPA, Manaus, Amazonas, Brazil; Onderstepoort Veterinary Institute, South Africa

## Abstract

The position of the Zoraptera remains one of the most challenging and uncertain concerns in ordinal-level phylogenies of the insects. Zoraptera have been viewed as having a close relationship with five different groups of Polyneoptera, or as being allied to the Paraneoptera or even Holometabola. Although rDNAs have been widely used in phylogenetic studies of insects, the application of the complete 28S rDNA are still scattered in only a few orders. In this study, a secondary structure model of the complete 28S rRNAs of insects was reconstructed based on all orders of Insecta. It was found that one length-variable region, D3-4, is particularly distinctive. The length and/or sequence of D3-4 is conservative within each order of Polyneoptera, but it can be divided into two types between the different orders of the supercohort, of which the enigmatic order Zoraptera and Dictyoptera share one type, while the remaining orders of Polyneoptera share the other. Additionally, independent evidence from phylogenetic results support the clade (Zoraptera+Dictyoptera) as well. Thus, the similarity of D3-4 between Zoraptera and Dictyoptera can serve as potentially valuable autapomorphy or synapomorphy in phylogeny reconstruction. The clades of (Plecoptera+Dermaptera) and ((Grylloblattodea+Mantophasmatodea)+(Embiodea+Phasmatodea)) were also recovered in the phylogenetic study. In addition, considering the other studies based on rDNAs, this study reached the highest congruence with previous phylogenetic studies of Holometabola based on nuclear protein coding genes or morphology characters. Future comparative studies of secondary structures across deep divergences and additional taxa are likely to reveal conserved patterns, structures and motifs that can provide support for major phylogenetic lineages.

## Introduction

**Figure 1:**
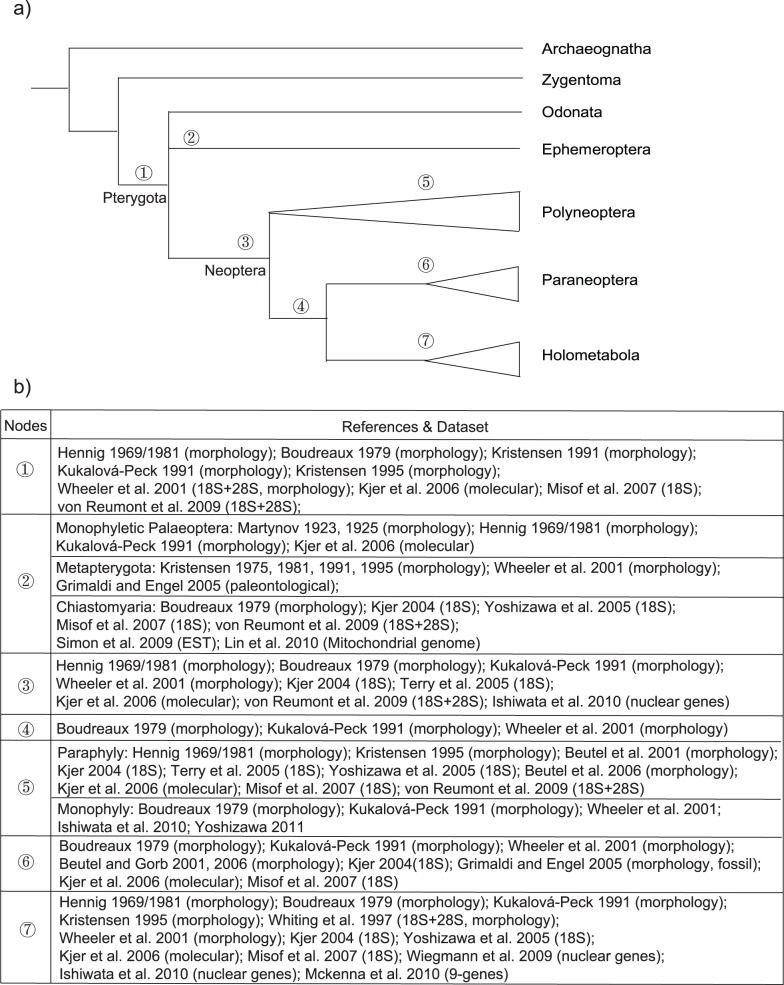
Currently recognized phylogeny of the Insecta. a) Summary cladogram, b) list of morphological and molecular studies.

Insects are the most diverse group of living organisms. The Insecta are comprised of the primitively wingless orders Archaeognatha (bristletails) and Zygentoma (silverfish), and the winged lineages of Odonata (dragonflies and damselflies), Ephemeroptera (mayflies), and the hyperdiverse Neoptera (all other insect lineages). The Neoptera themselves are divided into three large groups or supercohorts, the Polyneoptera, Paraneoptera, and Holometabola [1]–[6]. The currently recognized relationships between the orders of insects are summarized in Figure 1. With respect to the Neoptera, the monophyly of the Neoptera, Paraneoptera, and Holometabola have been overwhelmingly supported from morphological, paleontological, molecular, as well as combined analytical studies (Figure 1).

Compared to the Paraneoptera and Holometabola, there are many more uncertainties regarding the phylogenetic interrelationships, and even the monophyly as a whole, of the Polyneoptera. The Polyneoptera include the Dermaptera (earwigs), Plecoptera (stoneflies), Blattaria (roaches), Isoptera (termites), Mantodea (mantises), Zoraptera (angel insects), Notoptera [Grylloblattodea (ice crawlers) and Mantophasmatodea (rock crawlers)], Embiodea (webspinners), Orthoptera (crickets, grasshoppers, katydids, and wetas), and Phasmatodea (stick and leaf insects). Among these eleven orders, the monophyly of the Dictyoptera (Blattaria, Isoptera, and Mantodea) is the most widely accepted. In recent years, significant data from multiple sources has supported the unification of the Mantophasmatodea and Grylloblattodea as the order Notoptera [6], [7]–[13]. Of more controversy are the several studies uniting the stoneflies and earwigs [11], [14]–[16], although as noted by Yoshizawa [17] such a grouping is unstable and the Dermaptera remain challenging to place between the dictyopteran, plecopteroid, and orthopteroid orders.

Beyond the three clades mentioned above, there is virtually no consensus regarding the phylogeny of the Polyneoptera. The gaps between various taxonomic systems for the Polyneoptera are quite large (Table 1). The most considerable change in position remains that of the Zoraptera. This order is thought to be closely related either to Paraneoptera [4], [5], [18], [19], Holometabola [20], within Dictyoptera [15], [21], [22], with Dictyoptera+Dermaptera [23], Embiodea [6], [17], [24]–[27], Dermaptera [9], [12], or alongside Plecoptera+Dermaptera [28].

**Table 1:** Some traditional classifications of living orders in the Supercohort Polyneoptera.

Hennig (1953, 1969, 1981)	Blattopteroidea		Problematic “Orthopteroidea”		Remaining orders	
	Blattaria, Isoptera, Mantodea		Dermaptera, Grylloblattodea, Orthoptera, Phasmatodea		Plecoptera, Embiodea	
Sharov (1968)	Orthopteroidea				Remaining orders	
	Dictyoptera, Dermaptera, Grylloblattodea, Orthoptera, Phasmatodea				Plecoptera, Embiodea, Zoraptera	
Boudreaux (1979)	Orthopterodida				Remaining orders	
	Dictyoptera, Dermaptera, Grylloblattodea, Orthoptera, Phasmatodea, Zoraptera				Plecoptera, Embiodea	
Kristensen (1991, 1995)	Dictyoptera	Remaining orders				
	Blattaria, Isoptera, Mantodea	Dermaptera, Grylloblattodea, Orthoptera, Phasmatodea, Embiodea, Plecoptera				
Kukalová-Peck (1991)	Blattoneoptera	Orthoneoptera				Pleconeoptera
	Dictyoptera, Dermaptera, Grylloblattodea, Zoraptera	Orthoptera, Phasmatodea, Embiodea				Plecoptera
Grimaldi & Engel (2005), Arillo & Engel (2006)	Blattodea	Orthopterida		Dermapterida		Plecopterida
	Blattaria, Isoptera, Mantodea	Notoptera, Orthoptera, Phasmatodea		Dermaptera		Plecoptera, Embiodea, Zoraptera

Hennig [4], [5] and Kristensen [18], [29] placed the Zoraptera within the Paraneoptera based on their reduced numbers of tarsomeres, Malpighian tubes, and ganglia. However, the reduction of tarsomeres occurs independently in numerous insect lineages (e.g., Plecoptera, Dermaptera, Isoptera) and cannot be considered definitive evidence of relationship in isolation from other character sources. Subsequently, Minet and Bourgoin [24] allied the Zoraptera with the Embiodea based on a unique metatibial musculature, and Engel and Grimaldi [25], [26] expanded this suite of characters to include the loss of gonostyli, reduced cerci, enlarged metafemora, narrow and paddle-shaped forewings, presence of apterous morphs, dehiscent wings, and gregarious behavior. Yoshizawa [17], [30] later added numerous wing base characters to the list of synapomorphies supporting Zoraptera+Embiodea ( = Mystroptera). Others have argued that Zoraptera share some synapomorphies with the Dictyoptera, such as a highly derived flight motor, reduced phragmata, and greatly reduced indirect flight musculature [20], [21], but the latter two reductions occur homoplastically numerous times across many orders and cannot be considered stongly indicative of relationship. Simultaneously, molecular-based phylogenetic placements of the Zoraptera also have been uncertain [9], [12], [15], [28], [31] The main reasons for these controversies may be due to the analytical methods employed [14], [32]–[34] as well as the inclusion of dubious sequences [34], [35].

Ribosomal DNA sequences have been playing a major role in molecular phylogenetic studies in insects for the past two decades [36]. Analysis of 18S rDNA (also known as small subunit ribosomal DNA, SSU rDNA) has been used extensively in previous studies of insects at the ordinal level [9], [11], [12], [14], [15], [28], [31], [37]–[40]. However, analysis of 28S rDNA (also known as large subunit ribosomal rDNA, LSU rDNA) has not been employed in previous studies of insect phylogeny as generally as 18S has been. And in the cases of including 28S rDNA as one of the molecular markers, only partial segments, which vary from approximately 350 to 2000 nt, have been examined. It has been suggested that 28S rDNA contains significant phylogenetic signal for studying wide-ranging relationships [41]–[44]. Additionally, 28S rDNA shares many features with 18S rDNA, such as dramatic length variations, but is approximately two times the length of 18S rDNA and includes more variable regions, therefore representing a great suite of available data. The amplification of 28S rDNA is more likely interfered by the hairpin structures or tandem replicates of single nucleotides or oligonucleotides. As a result of these challenges, the application of complete 28S rDNA sequences in systematics has been hampered.

Among the aforementioned studies, only a few have employed information regarding the secondary structure of rRNAs [11], [12], [14], [15]. In fact, phylogenetic studies based on rDNAs can benefit considerably from information regarding rRNA secondary structure. First, the secondary structure of rRNA can be used to improve alignments and thus, improve the accuracy of tree construction [45]–[48]. The length variation in these sequences leads to ambiguous alignments, i.e., alternative arrangements of gaps. In addition, the hyper-length variation of some local regions may even result in incorrectly determined positional homology at a large scale [12]. For example, an length variable region (LVR) in the V4 region of 18S rDNA that does not exist in some species, such as *Pandinus imperator* (Arachnida, Scorpiones) [Genbank:AY210831] ranges up to a length of 1,349 nt in *Cubaris murina* (Crustacea, Isopoda) [Genbank:AJ287064] [49]. As length variation increases, it becomes increasingly difficult or even impossible to infer optimal alignment from multiple sequences using computational methods and manual procedures. Therefore, the alignment of sequences that include hyper-length-variable regions can be more problematic. Second, some LVRs can serve as synapomorphies for certain monophyletic groups. The members in a clade may share the same length or the same tendency for elongation of LVRs [12], [50].

A complete comparative study on the secondary structure of 18S rRNAs among insect orders was previously carried out by Gillespie et al. [51], Misof et al. [48], and Xie et al. [12]. For 28S rRNAs, there are two numbering systems for LVRs, which were alternatively referred to as D (divergent) domains [52], [53] or expansion segments [54], [55]. Twenty-two variable regions have been recognized for the major eukaryotic lineages [56], and some of these regions are hyper-length-variable regions. Among the insects, the complete or nearly complete secondary structures of 28S rRNAs have thus far been published for only a few species: *Drosophila melanogaster* (Diptera, Drosophilidae) [55], [56], *Aedes albopictus* (Diptera, Culicidae) [56], [57], *Acyrthosiphon pisum* (Hemiptera, Aphididae) [58], *Tenebrio* sp. (Coleoptera, Tenebrionidae) [59], *Apis mellifera* (Hymenoptera, Apidae) [60] and *Synthemis eustalacta* (Odonata, Synthemistidae) [61], and a comparative study of the full secondary structures of 28S rRNAs among all orders of insects is still lacking.

Herein, we examine the controversial position of Zoraptera utilizing complete 18S and 28S rDNA sequences in independent studies on secondary structure and phylogeny. In this study, we positioned all of the variable regions of the 28S rDNA sequences of insects and refined the boundaries of the conserved motifs based on the principles of co-variation [62]–[64] and compensatory or semi-compensatory substitution [65]. The regions of the 28S rDNA and 18S rDNA sequences with conserved lengths were used to reconstruct a phylogeny for Insecta with particular emphasis on the zorapterans, attempting to identify putative autapomorphies or synapomorphies for certain lineages.

## Materials and Methods

### Taxon Sampling

A dataset comprised of 28S and 18S rDNA sequences from 67 species was compiled representing all orders within the Insecta except for the Strepsiptera (Table S1), due to its only half sequenced length of 28S rDNA. The complete 28S rDNA sequences of 20 species and 18S rDNA sequences of 10 species were sequenced for the first time in this study. The newly acquired 28S rDNA sequences included two sequences for each of the orders Zoraptera, Archaeognatha, Thysanoptera, Neuroptera, and Psocoptera; one sequence from each of the orders Phthiraptera, Embiodea, Zygentoma, Megaloptera, and Trichoptera; and five sequences from Hemiptera.

### Molecular Experiments

Depending on the individual size of the sampled species, genomic DNA was extracted from either thorax tissue or the whole body except for the abdomen of ETOH-preserved insect specimens. Total genomic DNA was isolated using the CTAB-based method [66]. The primer sets used for amplification as well as sequencing were listed in Table S2. The functions of these primers were annotated in File S1. The PCR protocal for 28S rDNA included an initial denaturation at 94°C for 1 minute, followed by 30 cycles of 30 seconds at 94°C, 30 seconds-1 minute at 48–55°C and 1–2 minutes at 72°C, ending with a final extension at 72°C for 8–10 minutes. The thermal cycling program for 18S rDNA followed Johnson and Clayton [67]. All fragments were sequenced in both directions with the HiSeq 2000 sequencing system. A more detailed description of the molecular experiments was provided in the supplementary material File S1.

### Alignment and Phylogenetic Analysis

Sequence assembly was carried out using BioEdit 7.0 [68], and MEGA 5.01 [69], DAMBE 4.5.32 [70], [71], and Mesquite 2.75 [72] were used to align, connect and transform the format, respectively. Weblogo 3.0 was used to consent the sequences of expansion segment D3-4 [73], [74]. Reconstruction of secondary structure was realized by thermodynamic folding using RNAstructure 5.3 [75] and comparative methods [12], [45], [48], [54], [62]–[65], [76]. A more detailed description of the reconstruction of the secondary structure model was provided in the supplementary material File S1. Inkscape 0.48.2 was used for drawing the secondary structure (http://inkscape.org/). The secondary structure model of insect 18S rRNA followed published data [12]. The numbering system for LVRs of 28S rRNA followed the D system, which roughly includes thirteen D domains [52], [53]. All sequences were initially aligned using CLUSTAL X 2.0 software [77] and were then checked and corrected manually referring to the secondary structure models for 18S and 28S rRNAs. Nucleotides positions within which positional homology cannot be unambiguously aligned were eliminated during the process of phylogenetic reconstruction. The data matrix is attached as Dataset S1.

MrBayes 3.1.2 [78], [79] was used for Bayesian analysis. jModeltest 0.1.1 [80] was used to choose an appropriate model of substitution and GTR+G+I was selected as the best model for the data matrix. We used the parallelized version of MrBayes run on a graphics processing unit (GPU) [81] to speed up the calculation, achieving an approximately thirty times greater efficiency according to Nvidia GTX 580. The number of generations was 5,000,000, and the sampling frequency was 100. The average standard deviation of split frequencies fell below 0.01 after 1,901,000 generations, and the generations before generation 1,901,000 were burned-in. ML analysis was performed using Treefinder version 2011 [82]. The model GTR [Optimum, Empirical]: G [Optimum]:5 was determined by the program to be the best one. The number of bootstrap replicates was 1000. The other parameters were used with their default values.

## Results and Discussion

Complete 28S rDNA sequences of Zoraptera, Embiodea, Thysanoptera, Psocoptera, Phthiraptera, Neuroptera, and Megaloptera were provided for the first time in this study. These new data make each order of Insecta has at least one complete 28S rDNA sequence, except for Strepsiptera. These newly obtained 28S rDNA sequences make it possible to reconstruct the phylogeny of insects based on complete 18S and 28S rDNA sequences together. Besides, these complete sequences of 28S rDNAs also provide a background for further comparative studies of secondary structures of 28S rRNAs within each order of Insecta.

**Figure 2:**
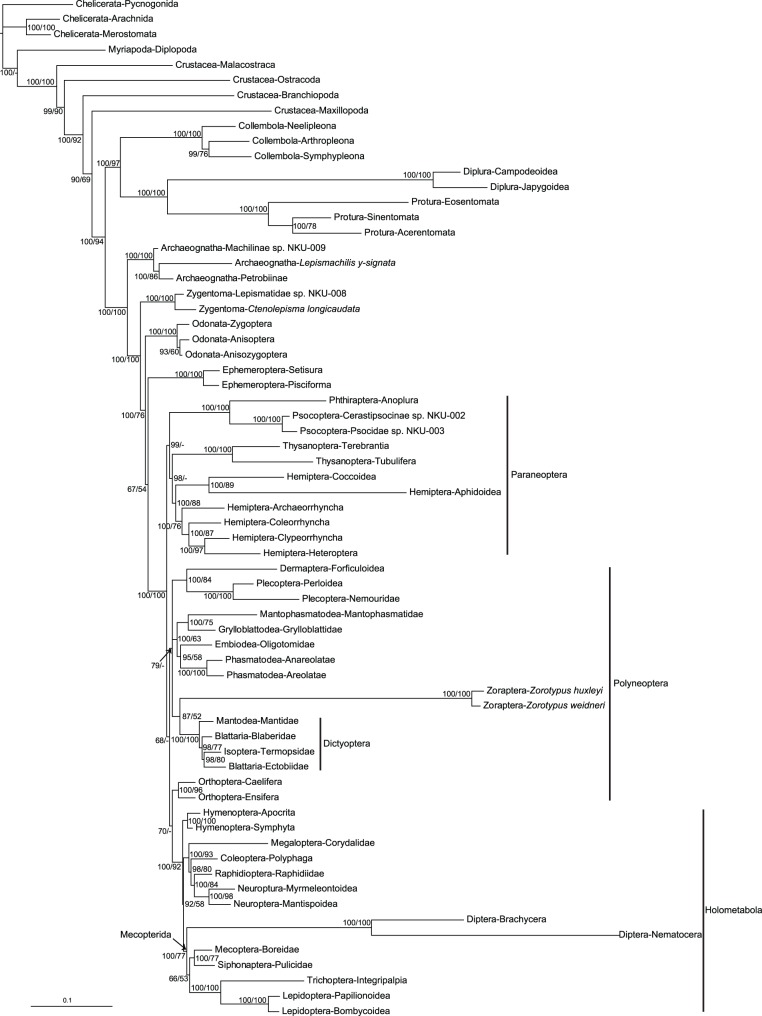
Phylogenetic tree inferred from the regions of the complete 18S and 28S rDNA sequences with conserved lengths. The numbers associated with the nodes are posterior probability values (first number) and bootstrap values (second number) obtained by Bayesian/ML analysis respectively. The lengths of the branches follow the phylogram of the Bayesian tree.

Both the monophyly of the Polyneoptera and the interrelationships within the Polyneoptera have been debated for quite some time. Results may vary based on the same type of data, such as morphological [4], [5], [19], [31], [83], [84] or rDNA data [12], [14], [15], [28] as well as between different types of evidence, such as mitochondrial and nuclear protein-coding gene (PCG) analyses [13], [85]. In recent years, EST [86], nuclear PCG [13], [87], [88], and mitochondrial genome sequences [85], [89] have been used to examine the order-level phylogeny of insects. Nevertheless, due to incomplete taxon sampling, incomplete data, and other hindrances, the positions of most orders within the Polyneoptera remain uncertain. In this study, the phylogenetic results based on the length-stable regions of complete rDNAs are summarized in Figure 2. The consensus tree simultaneously recovered many clades indicated separately in various studies, such as Notoptera (which include the Mantophasmatodea and Grylloblattodea) [8]–[10], Plecoptera and Dermaptera [11], [14], [15], [17], Embiodea and Phasmatodea [9], [13], [29], [90], and Condylognatha (which include the Hemiptera and Thysanoptera) [4], [5], [13], [15], [21], [28], [91].

### Position of the Zoraptera

The clade (Zoraptera+Dictyoptera) received a posterior probability of 87% in the BI analysis and a bootstrap value of 52% in the ML analysis. This bootstrap value, and support in general, is rather low. However, the same topology exists in both the BI and ML results. The low support value may be due to the unique evolutionary patterns among the Zoraptera, such as an accelerated substitution rate and unique insertions and deletions (indels), as have been noted in studies using 18S rDNA as a molecular marker [11], [15]. The special attributes of the Zoraptera can be observed in the BI results (Figure 2), in which the Zoraptera lineage is a rather long branch. The distinctive quality of Zoraptera rDNAs may be one of the reasons for the disputed status of this group in previous studies.

**Figure 3:**
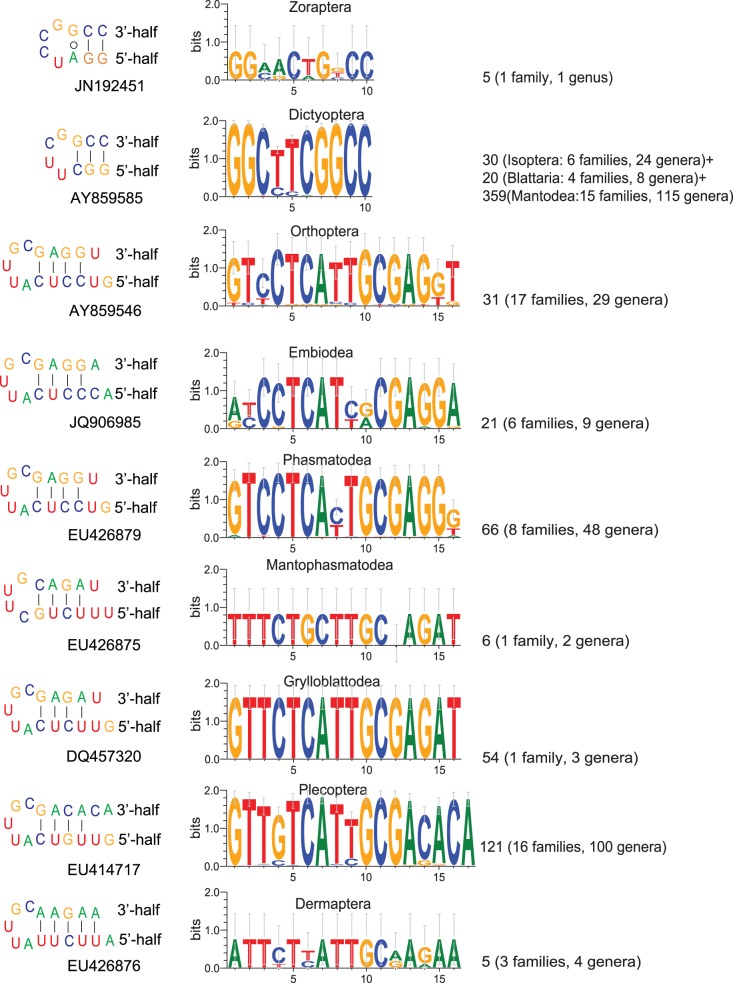
Consensus of the sequences of expansion segment D3-4 based on the homologues in GenBank. The left column is the secondary structure of the D3-4 ‘marker-box’ of each polyneopteran order. The accession number in the bottom of each regional secondary structure stands for the corresponding sequence which is the same to the consensus sequence of each polyneopteran order or superorder. The middle column is the consensus result of the homologues in each polyneopteran order or superorder. The abscissa stand for the number of the bases, while the ordinate stand for the proportion of information content provided by each base in the same position. The right column is the number of sequences based on.

**Figure 4:**
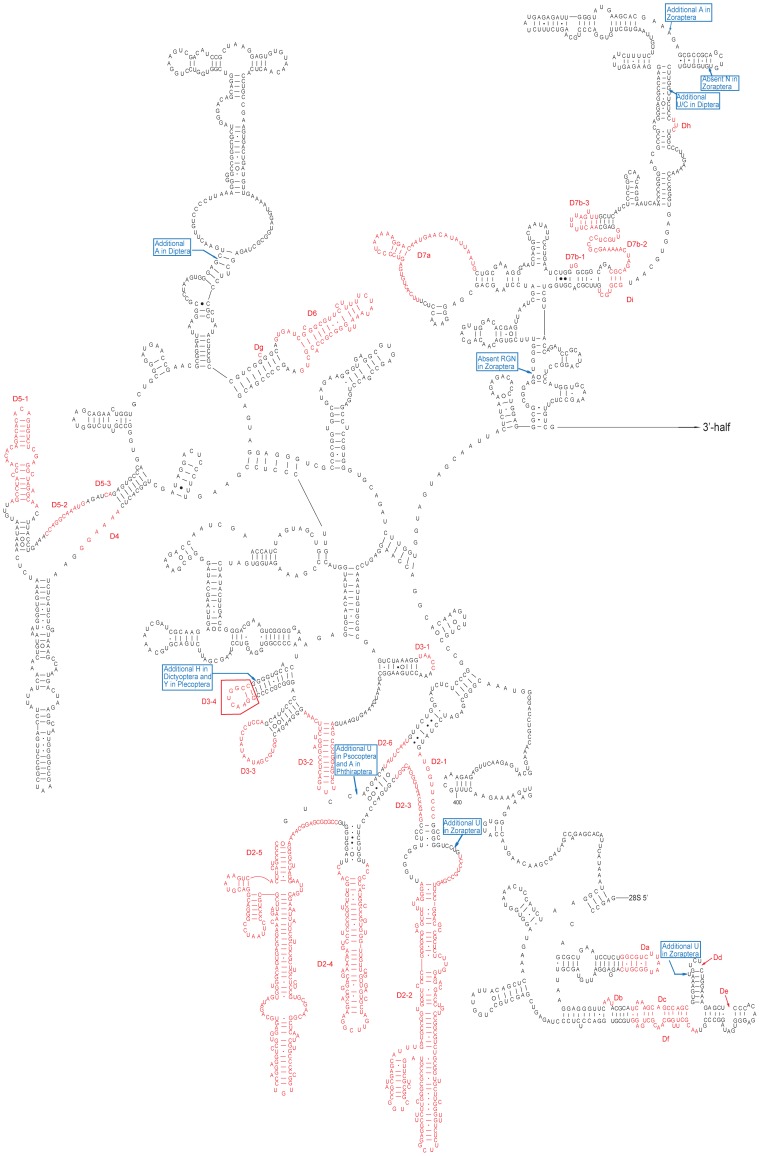
Secondary structure model of the 28S rRNA 5′-half of Zoraptera. This sequence is from *Zorotypus huxleyi* [Genbank:JN192451]. The length-variable regions are indicated in red. And the unique indels are marked with green color. The D3-4 box was highlighted with thick red lines. The Da–Dj numbering system for LVRs, which has not been taken into account previously, is a supplementary system to the D1–D12 coding system. Base pairing is indicated as follows: standard canonical pairs by lines (C-G, G-C, A-U, U-A); wobble G·U pairs by dots (G·U); A·G and A·C pairs by open circles (A G, A C); other non-canonical pairs by filled circles (e.g., U•U).

In the present study, independent secondary structure evidence also supports a sister relationship between the Dictyoptera and Zoraptera (Figure 3). Among all of the detected LVRs, we found that D3-4 is particularly special. The length and/or sequence of D3-4 is conservative in each order of Polyneoptera, but it can be divided into two types among the different orders of the supercohort (Figure 3). The Zoraptera and Dictyoptera shared one type of 10 nucleotides length, and the Plecoptera, Dermaptera, Orthoptera, Phasmatodea, Embiodea, Grylloblattodea, and Mantophasmatodea shared the other type of 16±1 nucleotides length. There is a unique insertion special for Plecoptera around 3′-end, and there is a unique deletion of G special for Mantophasmatodea near the 3′-end. The length differences between the 10 nucleotides and the 15–17 nucleotides are extremely significant (File S1). This attribute makes D3-4 a good marker to indicate relationships within Polyneoptera. The clade (Zoraptera+Dictyoptera) shares a unique 10 nt long box in this expansion segment, and the D3-4 sequences of these two groups are also similar, i.e., GGYYYMKGCC in Dictyoptera and GGMRCWGBCC in Zoraptera. In Figure 4, if considering there is only one base pair between the D3-4 and the single insertion in some groups, these two parts can be alternatively viewed as a whole. However, even if this possibility is taken into account, the length and sequence of D3-4 is still most similar to those of Dictyoptera. Therefore, this alternative consideration on the boundary of D3-4 has no bias according to the competitive hypotheses of the phylogenetic position of Zoraptera.

Length-stable and length-variable rDNA regions are under different evolutionary constraints [46]. Most LVRs are distributed on the surface of the tertiary structure of rRNA, far from the functional site [49]. Although weak constraints of negative selection might also lead to homoplastic patterns in non-related taxa, the extremely significant differences between the lengths and similarity of the sequences make the probability quite low that the same pattern of D3-4 has originated independently in Zoraptera and Dictyoptera. Accordingly, a Zoraptera+Dictyoptera clade may have some credence if corroborated by future analyses and other forms of data. Besides the significance in understanding the evolutionary history of hexapods, resolving the phylogenetic position of Zoraptera is also very important because the resolving influences findings regarding shifts in diversification of hexapods [92].

The Dictyoptera represent the most universally accepted supra-ordinal grouping within the Polyneoptera. The three orders of Dictyoptera share several synapomorphies, including an extremely reduced ovipositor, mostly internal valvulae, perforated tentorium, and asymmetrical male genitalia [16], [21], [93]. At the molecular level, the length of one LVR in the 18S rRNA secondary structure was discovered to represent a synapomorphy of the Dictyoptera [12]. In the comparative analysis of 28S rRNA, a unique length of expansion segment D4 of 14 nt was found to be shared by all three orders of Dictyoptera and, thus, constitutes a further molecular autapomorphy for Dictyoptera. Additionally, the three orders of Dictyoptera exhibit the same lengths for another 18 out of 40 LVRs.

The reduction of the ovipositor is a highly homoplastic character which occurs innumerably and independently across Polyneoptera, within orders, within families, sometimes even within a single genus, and, indeed, across all Insecta. Similarly, “mostly internal valvulae” also occurs many times independently. Additionally, Zoraptera do not have a perforated tentorium, and male genitalia are symmetrical. At present there is no compelling morphological evidence for a Zoraptera+Dictyoptera clade, and it will require future testing by expanded data sets.

### Position of the Embiodea

Among the Polyneoptera, Embiodea is another order for which the position remains controversial. From a morphological perspective, Embiodea was hypothesized to be either a sister group of Plecoptera [21], [31], Phasmatodea [18], [29], [90], [93]–[96], Dermaptera [84], or Zoraptera [6], [17], [24]–[27]. However, in the past several years, most results based on molecular data support the Embiodea and Phasmatodea hypothesis [9], [11], [13], [97]. The phylogenetic results of this study based on complete 18S and 28S rDNA sequences also support these two orders as closely related (Figure 2). Although the bootstrap value for Embiodea+Phasmatodea is low in this analysis, it is congruent with the Bayesian result of this study and the results of other molecular phylogenetic studies. In addition, these analyses supported the sister relationships between Grylloblattodea and Mantophasmatodea. These two monophyletic groups further formed a clade, which is congruent with Kjer et al. [11].

The clade ((Grylloblattodea+Mantophasmatodea)+(Embiodea+Phasmatodea)) was supported with high posterior probability values (100%). A clade including Grylloblattodea, Phasmatodea and Embiodea has been suggested based on 18S rDNA sequences [15], but without sampling Mantophasmatodea. Similarly, a clade including Grylloblattodea, Mantophasmatodea, and Phasmatodea has recently been indicated based on mitochondrial genomes [85], but without sampling Embiodea. This superordinal grouping, here called the “Mecynoptera hypothesis”, is novel to our study and deserves critical investigation. It is unclear to what extent this grouping may or may not be supported by existing paleontological data.

Based on morphological studies addressing fossils or living groups, Orthoptera had been viewed to have a close relationship with Phasmatodea [19], [21], [31], [98]–[101]. However, in molecular phylogenetic studies, the position of Orthoptera has often been indeterminate [9], [11], [13]–[15], [28], [85]. In our study, the position of Orthoptera is shown as unresolved in the results of both the BI and ML analyses. However, Orthoptera share the same length and similar sequences of D3-4 with the Mecynoptera clade. Thus, these five orders may constitute a potential group, with Orthoptera as basal, but the phylogenetic signal in the available rDNA sequences is not sufficiently strong to make a definitive conclusion.

### Taxonomy of Polyneoptera

Handlirsch [102] first suggested the existence of two subclasses of Polyneoptera: Orthopteroidea and Blattaeformia. Subsequently, due to changes in hierarchical systems of subclasses, infraclasses, or superorders, the name Orthopteroidea has come to have different meanings for different researchers (Table 1). Orthopteroidea can indicate a group as small as consisting of only the Orthoptera, Phasmatodea, and Embiodea [96], or as large as including all of the Polyneoptera [9], [31], [103]. According to the results of this study, we tentatively suggest that there might be recognized a revised Dermoplecopterida for (Plecoptera+Dermaptera), the Blattopterida as (Dictyoptera+Zoraptera), and the Mecynoptera equal to the ((Embiodea+Phasmatodea)+(Grylloblattodea+Mantophasmatodea)).

The relationships between these four putative lineages, i.e., Dermoplectopterida, Blattopterida, Orthoptera, and Mecynoptera, are not effectively resolved in this study. In the future, combining rDNA results with analysis of nuclear PCGs may contribute to completely resolving the phylogeny of the Polyneoptera. It is also possible that the unresolved nodes within the Polyneoptera may due to ancient rapid radiation [104]–[106], as rapid diversification would result in particularly short inter-divergence times within which characters could accumulate. In fact, even phylogenomic studies based on EST may include unresolved or weakly supported nodes [105]–[114].

### Phylogeny of the Eumetabola

The Paraneoptera are comprised of the Psocoptera (book lice and bark lice), Phthiraptera (lice), Hemiptera (true bugs), and Thysanoptera (thrips). Psocoptera and Phthiraptera are together referred to as the superorder Psocodea [115], and the monophyly of the Psocodea has been supported by numerous studies [11], [13], [14], [28], [116]. According to the phylogenetic relationships within the Paraneoptera, most morphological studies consistently view Thysanoptera as the sister group to Hemiptera, and these two orders are referred to as the superorder Condylognatha [4], [5], [21], [29], [31], [91], [116]. Compared to results based on morphological data, results based on molecular analyses may differ from each other. Among results from 18S rDNA analyses, the position of Thysanoptera has been shown to be close to either Psocodea [31] or Hemiptera [28]. In studies based on multiple nuclear genes, Thysanoptera was shown to be close to Hemiptera, supported by a high posterior probability in BI [13], [15]. In this study, the monophyly of the Condylognatha was confirmed with a high probability in the Bayesian inference. Thus, the monophyly of the Condylognatha is now supported by evidence from morphological, nuclear PCG, and rDNA analyses.

Within the Holometabola, the phylogenetic results strongly supported a basal position for the Hymenoptera. The other orders of Holometabola are further segregated into two principal clades: Mecopterida ( = Diptera+(Mecoptera+Siphonaptera)+(Trichoptera+Lepidoptera)); and Coleoptera+Neuropterida ( = Megaloptera+Raphidioptera+Neuroptera) (Figure 2). This may be the first time that evidence from rDNAs has been consistent with that from nuclear PCGs [13], [87], [88], [117]. In fact, if the partially sequenced rDNAs of Strepsiptera, as mentioned in the part Taxon Sampling of Material and Methods, are included in the taxon sampling of this study, its phylogenetic position is the sister group to Coleoptera in the Bayesian tree (Figure S1). This would make the phylogenetic result of Holometabola part more congruent with the result based on morphology [118] or other molecular markers [13], [87], [88], [117], [119], [120].

### The Impact of LVRs on Alignment and Phylogeny

**Figure 5:**
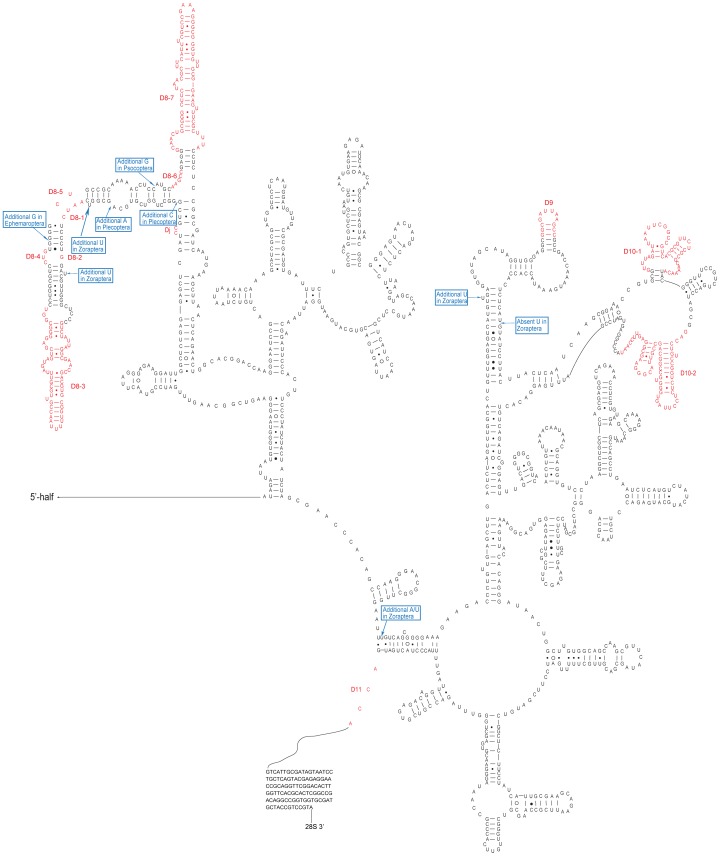
Secondary structure model of the 28S rRNA 3′-half of Zoraptera. This sequence is from *Zorotypus huxleyi* [Genbank:JN192451]. The length-variable regions are indicated in red. And the unique indels are marked with green color. The Da–Dj numbering system for LVRs, which has not been taken into account previously, is a supplementary system to the D1–D12 coding system. Base pairing is indicated as follows: standard canonical pairs by lines (C-G, G-C, A-U, U-A); wobble G·U pairs by dots (G·U); A·G and A·C pairs by open circles (A G, A C); other non-canonical pairs by filled circles (e.g., U•U).

A general secondary structure model for insect 28S rRNA was reconstructed (Figures 4 and 5, Figures S2 and S3), and there were a total of 40 LVRs detected. Most of the LVRs consisted of single strands located around lateral or terminal bulges (Figures 4 and 5), while the others were internal bulges or multi-branched loops. The length variation of each LVR was summarized in Tables S3 (18S) and S4 (28S). According to the extent of length variability for each LVR of 28S rDNA, D2, D3, D5, D7, D8, and D10 were the most extensive LVRs or hyper-variable regions (Table S4). Variations in these six expansion segments accounted for approximately 87.5% of the total variability among all LVRs. Among the expansion segments, D8-3 was the most variable, ranging from 2 nt in Diptera [Genbank:L78065] to 524 nt in Neuroptera [Genbank:JQ259053]. Among the investigated groups, Phthiraptera [Genbank:JQ309932] and Strepsiptera [Genbank:HM156704] exhibited the most extensive LVRs of 28S rDNA.

The accuracy and quality of rDNA alignments are critical factors in molecular phylogenetic studies [14], [12], [45], [47], [50], [56], [121]–[123]. Nucleotide positions for which positional homology cannot be unambiguously or correctly aligned should be eliminated during the process of phylogenetic reconstruction [14], [45], [50]. In this work, the impact of LVRs on the alignment and, thus, on the phylogeny was considered. The tree obtained from the automatic alignment results for the combined 18S and 28S rDNA sequences (Dataset S2, Figures S4 and S5) yielded results presenting many contradictions compared to widely accepted opinions. For example, Dermaptera is imbedded within Holometabola; Archaeognatha is the sister group of Odonata; the Holometabola are paraphyletic; and Coleoptera and Ephemeroptera are sister groups. Therefore, in this case study, the comparative phylogenetic results reinforce the opinion that due to the improved alignment, the performance of rDNA regions with conserved lengths can be superior to that of the original sequences (Figure 2, Figures S4 and S5). However, it is imperative that the delimitation of conserved and variable regions be ascertained. Otherwise, more informative sites will be lost during the abandonment of ambiguous regions.

In this study, the secondary structure model of 28S rRNA reconstructed for eukaryotes [56], [76] was specifically refined for insects. For a specific taxon, the hyper-variable regions summarized for eukaryotes can be divided into several small regions. After the comparative analysis of insect 28S rRNAs, six hyper-variable regions in the secondary structure model for eukaryotes were split into a number of sub-regions. For example, the hyper-variable region D8 was divided into eight sub-regions in this study (Figure 5). With respect to the phylogeny of the lower categories, these sub-regions can be further subdivided [59], [121]. Group-specific analysis will be helpful in exploring possible evidence of common origins based on the secondary structure of rRNA. The expansion segment D3-4 of 28S rRNA is such a case.

In this study, the substitution models of base pairs were not applied. The use of specific mixed RNA/DNA substitution models in insect rRNA phylogenetics might not lead to more reasonable results, most likely due to substitutional saturation in unpaired regions [123], [124]. In addition, compared to the biological background of structural studies of macromolecules, the substitution models of base pairs provided by current phylogenetic programs are not complete. Generally, current substitution models treat base pairs as only three different types, the canonical base pairs, GU-UG pairs, and all of the other modes of base pairs. The AC/CA and AG/GA base pairs, which exist subjectively in three dimensional structures of rRNAs [125], are not viewed as regular base pairs by current phylogenetic programs. The improvement of substitution models of base pairs in the future deserves being tested further.

### Conclusions

The results of this work provided novel evidence to support the close relationship between Zoraptera and Dictyoptera from the views of secondary structure and phylogeny independently. Besides, the present analysis first provided the direct evidence to support the monophyly of the clade ((Embiodea+Phasmatodea) + (Grylloblattodea+Mantophasmatodea)). The results of this work also reached the highest congruence with the results of previous molecular phylogenetic studies of insects based on nuclear PCGs, especially those of Holometabola. Accordingly, these results for understanding the higher-level relationships and diversification of insects are of critical importance.

This study can also serve as one more case to support that, the LVRs can remarkably affect the result of alignment, and thereby the result of phylogeny. Based on the secondary structure model of the 28S rRNA reconstructed in this study, all of the LVRs were removed *a priori* and the complete rDNAs were aligned unambiguously. Due to the improved alignment, the performance of rDNA regions with conserved lengths can be superior to that of the original sequences.

## Supporting Information

Figure S1 **Bayesian tree inferred from analysis of the complete 18S and 28S rDNA sequences with conserved lengths.** The sequences of Strepsiptera were included in this phylogenetic analysis. The number of generations was 10,000,000, the sampling frequency was 100, and the first 7,060,000 generations was discarded as “burnin”. This is a majority rule consensus tree, and the Bayesian posterior probability is given above each corresponding node.
(JPG)

Figure S2 **Secondary structure model of the 28S rRNA 5′-half of Insecta.** This sequence is from *Drosophila melanogaster* [GenBank:M21017]. The length-variable regions are indicated in red. And the unique indels are marked with green color. The D3-4 box was highlighted with thick red lines. Base pairing is indicated as follows: standard canonical pairs by lines (C-G, G-C, A-U, U-A); wobble G·U pairs by dots (G·U); A·G and A·C pairs by open circles (A G, A C); other non-canonical pairs by filled circles (e.g., U•U).
(JPG)

Figure S3 **Secondary structure model of the 28S rRNA 3′-half of Insecta.** This sequence is from *Drosophila melanogaster* [GenBank:M21017]. The length-variable regions are indicated in red. And the unique indels are marked with green color. Base pairing is indicated as follows: standard canonical pairs by lines (C-G, G-C, A-U, U-A); wobble G·U pairs by dots (G·U); A·G and A·C pairs by open circles (A G, A C); other non-canonical pairs by filled circles (e.g., U•U).
(JPG)

Figure S4 **Tree obtained by Bayesian analysis of the complete 18S+28S rDNAs.** The rDNA sequences were aligned by Cluxtal X, not adjusted by manual according to the secondary structures of the rDNAs. The number of generations was 5,000,000, the sampling frequency was 100, and the first 364,000 generations was discarded as “burnin”. This is a majority rule consensus tree, and the Bayesian posterior probability is given above the node.
(JPG)

Figure S5 **ML tree based on the automated alignment result of complete 18S+28S rDNAs.** Numerals above the nodes are bootstrap values.
(JPG)

Table S1 **Taxa sampling of 18S and 28S rDNAs.** Accession numbers marked with an asterisk are newly sequenced for 28S rDNAs (and for 18S rDNAs when needed) in the present study. The sequences of Strepsiptera were not included in the phylogenetic analyses, but in the study of secondary structure rRNAs.
(XLS)

Table S2 **Primer sets used for amplification and sequencing.** The primer sets of DF1-FD1 and EE-GG were newly designed in this study and can be used universally for insects. The rest were specifically designed for some groups.
(XLS)

Table S3 **The detailed information of the variable regions of the 18S rDNA for each taxon.**
(XLS)

Table S4 **The detailed information of the variable regions of the 28S rDNA for each taxon.**
(XLS)

Dataset S1 **The data matrix of the conservative parts of the 18S and 28S rDNA.** This matrix only included the conservative region of the combined 18S and 28S rDNA. The variable regions were excluded in advance referred to the secondary structure of the 18S and 28S rRNA respectively.
(FAS)

Dataset S2 **The data matrix of the combined 18S and 28S rDNA.** This matrix was generated through alignment programs, which included the complete 18S and 28S rDNAs.
(FAS)

File S1 **The detailed annotation of methods.**
(DOC)
