# Correction: A Unique Box in 28S rRNA Is Shared by the Enigmatic Insect Order Zoraptera and Dictyoptera

**DOI:** 10.1371/annotation/4bf59d03-84aa-45af-bf23-57e068beaaec

**Published:** 2013-10-11

**Authors:** Yanhui Wang, Michael S. Engel, Jose A. Rafael, Kai Dang, Haoyang Wu, Ying Wang, Qiang Xie, Wenjun Bu

The published Figure 3 is incorrect. Please view corrected Figure 3 here: 

**Figure:**
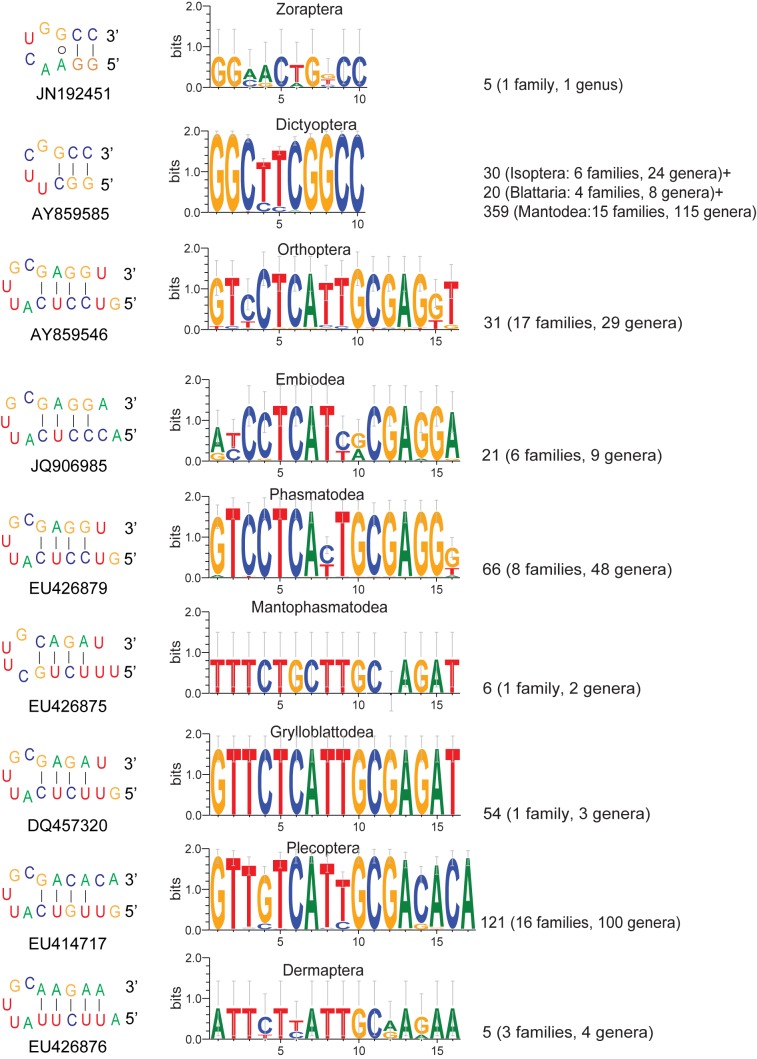


There was an error with the first affiliation for author Michael S. Engel. The correct first affiliation is:

Division of Entomology (Paleoentomology), Natural History Museum, University of Kansas, Kansas, USA 

