# Supplementary figures and images for: A Unique Box in 28S rRNA Is Shared by the Enigmatic Insect Order Zoraptera and Dictyoptera
Source: PLoS One. 2013 Jan 3;8(1):e53679. doi: 10.1371/journal.pone.0053679 (PMC3536744; doi:10.1371/journal.pone.0053679)

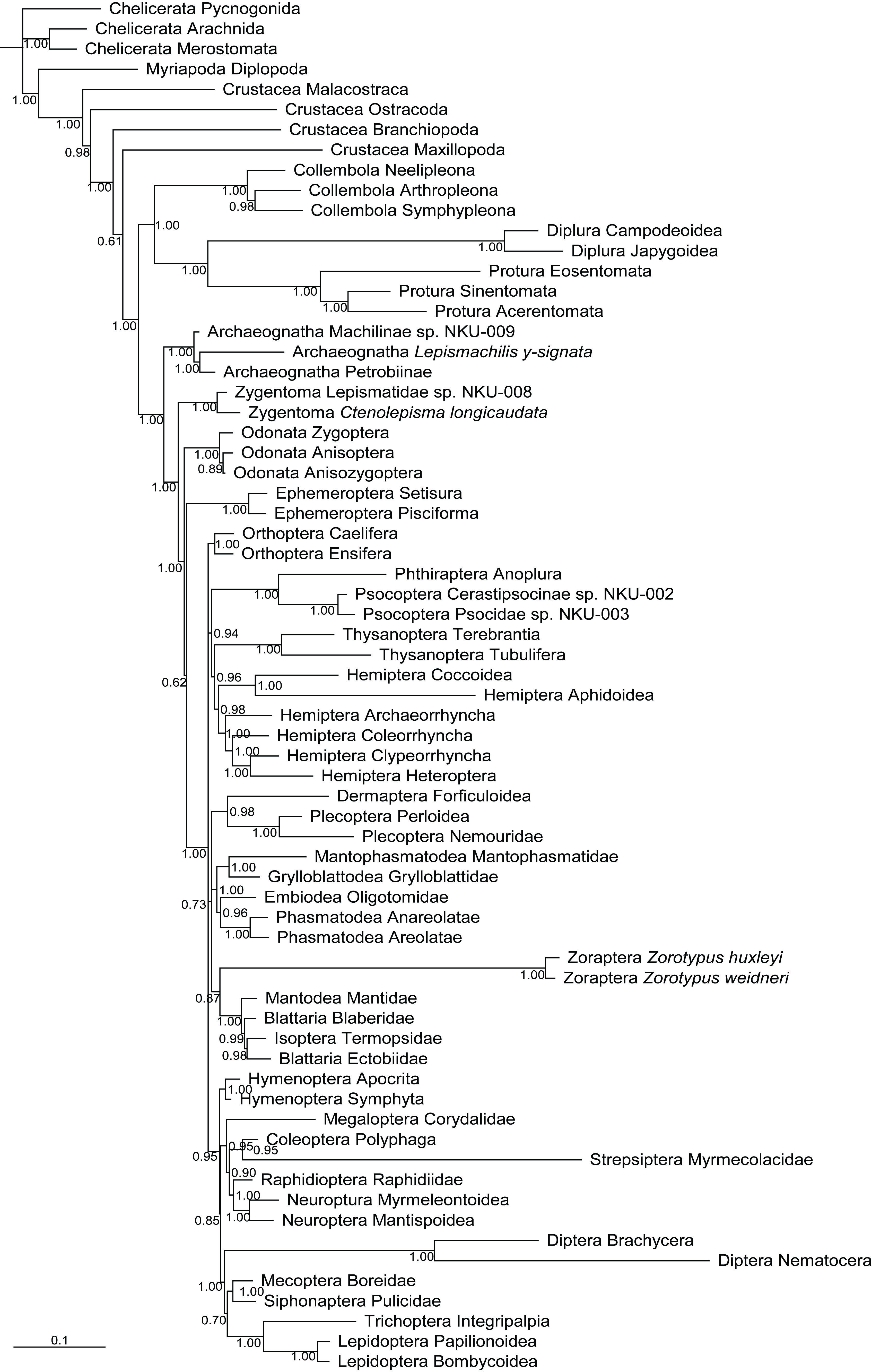

Supplement: Figure S1 — Bayesian tree inferred from analysis of the complete 18S and 28S rDNA sequences with conserved lengths. The sequences of Strepsiptera were included in this phylogenetic analysis. The number of generations was 10,000,000, the sampling frequency was 100, and the first 7,060,000 generations was discarded as “burnin”. This is a majority rule consensus tree, and the Bayesian posterior probability is given above each corresponding node. (JPG) [file pone.0053679.s001.jpg]

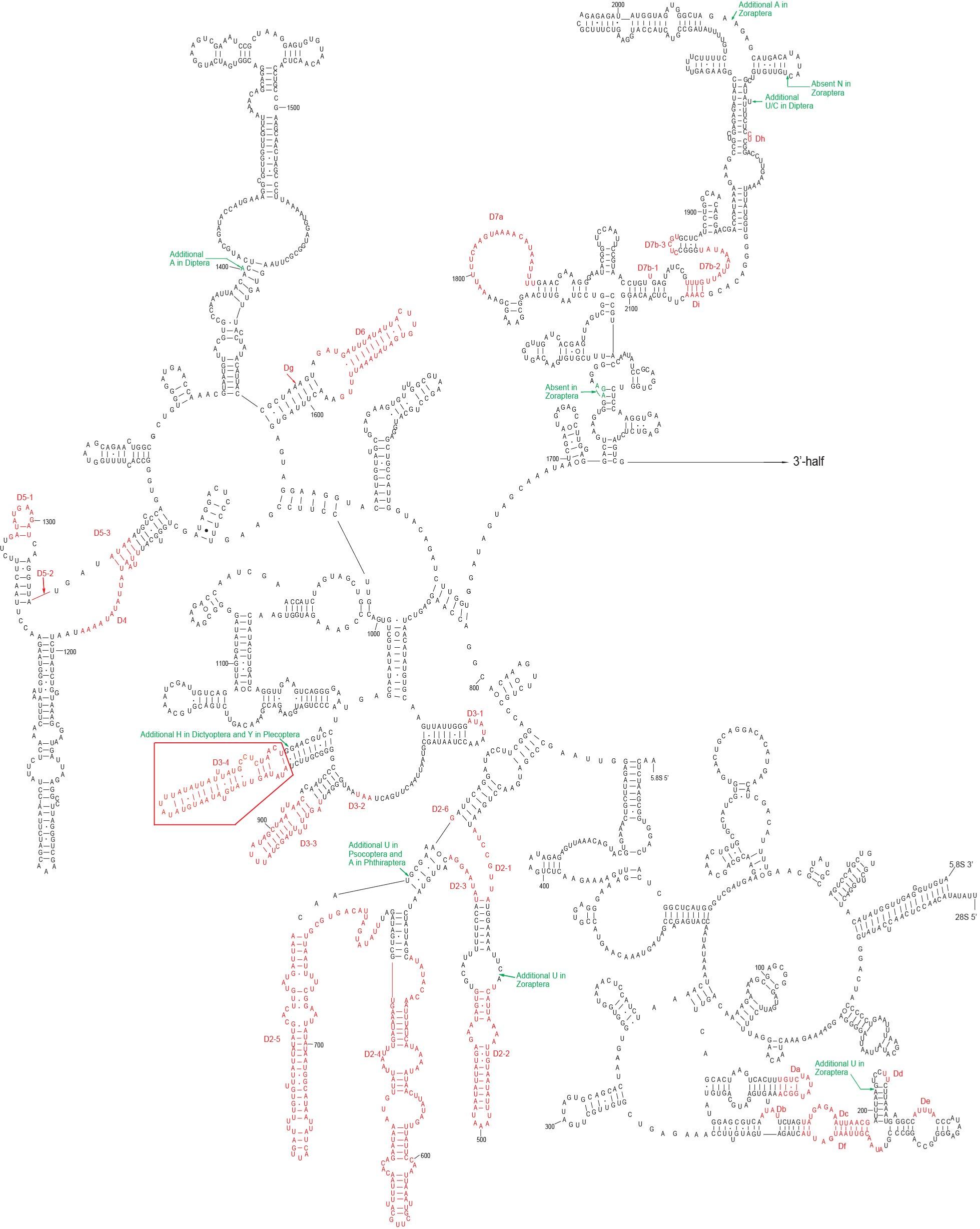

Supplement: Figure S2 — Secondary structure model of the 28S rRNA 5′-half of Insecta. This sequence is from Drosophila melanogaster [GenBank:M21017]. The length-variable regions are indicated in red. And the unique indels are marked with green color. The D3-4 box was highlighted with thick red lines. Base pairing is indicated as follows: standard canonical pairs by lines (C-G, G-C, A-U, U-A); wobble G·U pairs by dots (G·U); A·G and A·C pairs by open circles (A G, A C); other non-canonical pairs by filled circles (e.g., U•U). (JPG) [file pone.0053679.s002.jpg]

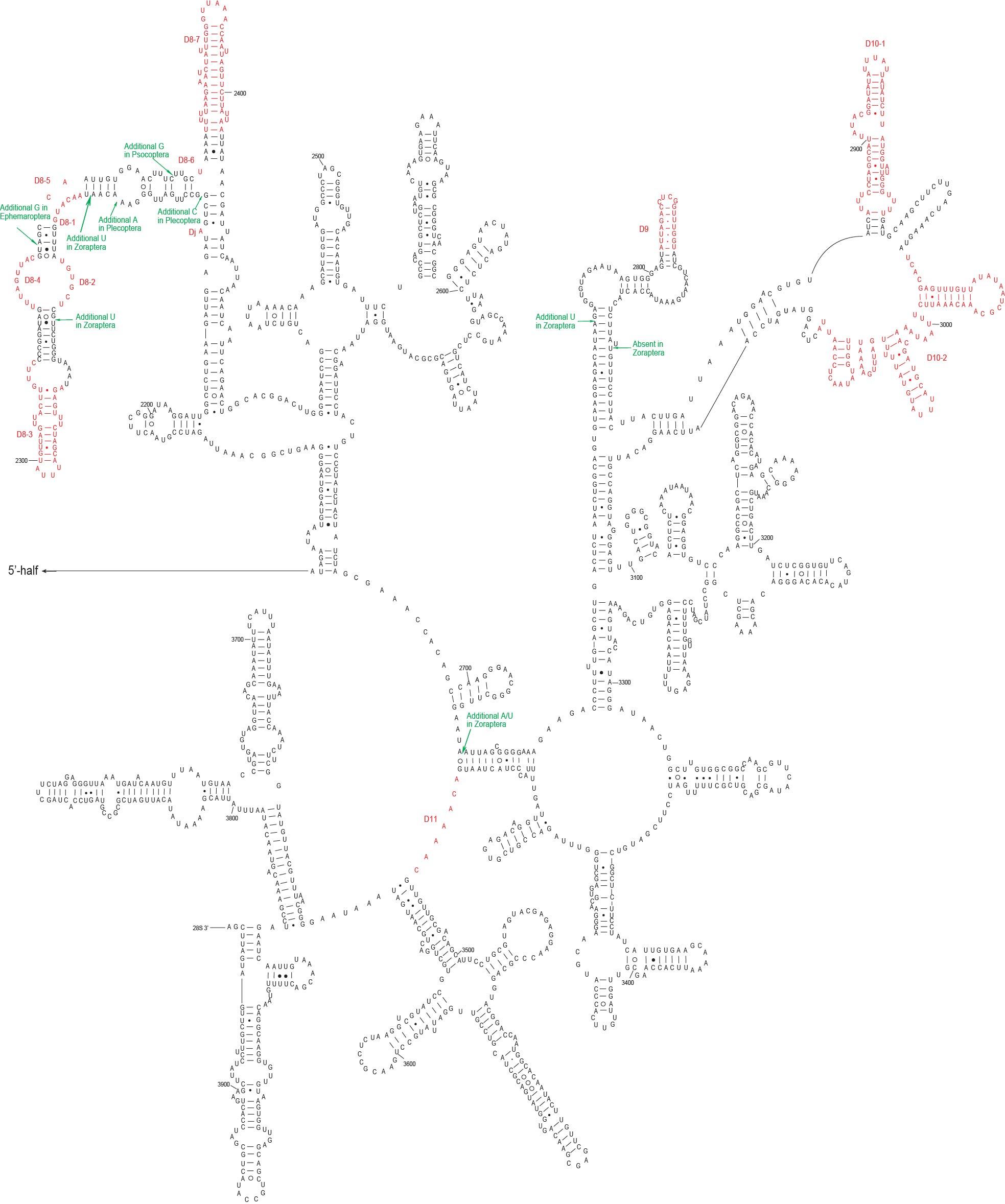

Supplement: Figure S3 — Secondary structure model of the 28S rRNA 3′-half of Insecta. This sequence is from Drosophila melanogaster [GenBank:M21017]. The length-variable regions are indicated in red. And the unique indels are marked with green color. Base pairing is indicated as follows: standard canonical pairs by lines (C-G, G-C, A-U, U-A); wobble G·U pairs by dots (G·U); A·G and A·C pairs by open circles (A G, A C); other non-canonical pairs by filled circles (e.g., U•U). (JPG) [file pone.0053679.s003.jpg]

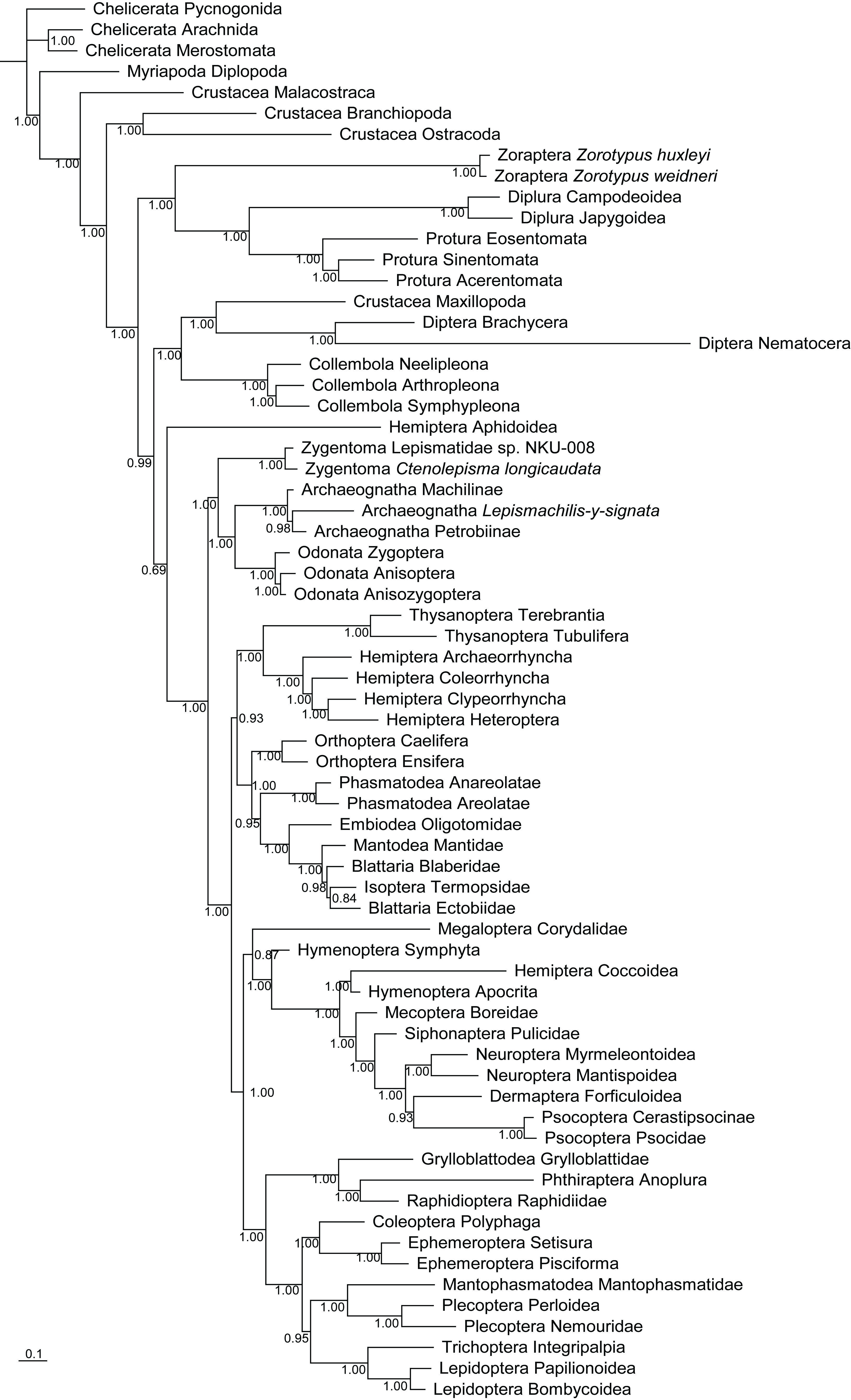

Supplement: Figure S4 — Tree obtained by Bayesian analysis of the complete 18S+28S rDNAs. The rDNA sequences were aligned by Cluxtal X, not adjusted by manual according to the secondary structures of the rDNAs. The number of generations was 5,000,000, the sampling frequency was 100, and the first 364,000 generations was discarded as “burnin”. This is a majority rule consensus tree, and the Bayesian posterior probability is given above the node. (JPG) [file pone.0053679.s004.jpg]

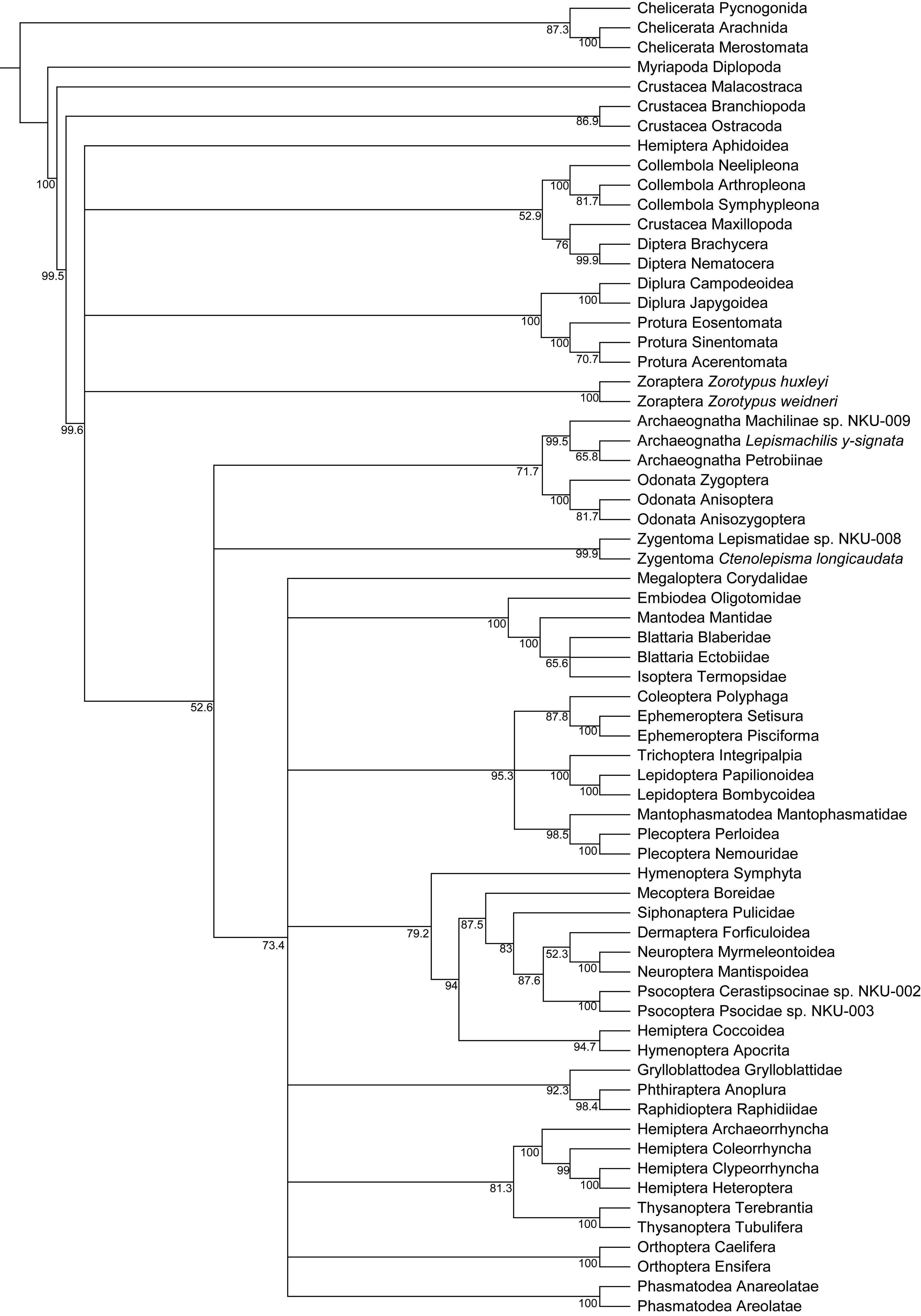

Supplement: Figure S5 — ML tree based on the automated alignment result of complete 18S+28S rDNAs. Numerals above the nodes are bootstrap values. (JPG) [file pone.0053679.s005.jpg]
