# Supplementary material for: A Unique Box in 28S rRNA Is Shared by the Enigmatic Insect Order Zoraptera and Dictyoptera
Source: PLoS One. 2013 Jan 3;8(1):e53679. doi: 10.1371/journal.pone.0053679 (PMC3536744; doi:10.1371/journal.pone.0053679)
Supplement: File S1 — The detailed annotation of methods. (DOC) [file pone.0053679.s012.doc]

**Method to draw secondary structure**

The secondary structure model for the 28S rRNA of Hexapoda is modified from the general secondary structure model of eukaryotic 28S rRNA [1-2]. The length conservative parts were kept while each of the length variable regions was refolded using RNAstructure 5.3. For a certain length variable region, the secondary structures of all homologues were compared and the majority consensus structure, i.e. the structure which appeared higher than 50%, was used as the model of that length variable region. If no competitive structure appeared higher than 50%, the structure of that region was left as the length variable region. When using RNAstructure 5.3, the parameters were set with the default values. Compensatory or semi-compensatory substitution can help to verify the paired regions.

**Molecular experiments**

The primer sets used for amplification as well as sequencing were listed in File S2. These primer sets were used to amplify six overlapping fragments of 28S rDNA and three overlapping fragments of 18S rDNA. The primer sets DF1-FD1 and EE-GG were designed in this study and can be used universally for insects. The primer sets Ns1-Ns2a and Ns5a-Ns8 were from Barker et al. [3], while 18Sai-18Sbi was from Whiting et al. [4], and Ns5aP2-Ns8P was from Johnson et al. [5]. Additional specific primer sets were specifically designed for some groups.

PCR amplifications were performed in 50 µl volumes containing 33.7 µl of ddH2O, 8 µl of dNTP (2.5 mM), 5 µl of 10×buffer (Mg 2+ Plus), 1 µl of primer each (10 µM), and 0.3 µl of Taq (5 U). PCR products were inspected in 0.8-1.2% agarose gels through electrophoresis, then purified using an EZ-10 column DNA Gel Extraction Kit according to the manufacturer's recommendations. Fragments with complex structures, such as tandem replicates of nucleotides, were sequenced by cloning using the pEASY-T3 Cloning Kit following the manufacturer's instructions.

**Statistical analysis of the lengths differences of the D3-4 region**

We use sample_1_ to represent the Polyneoptera group which excludes (Zoraptera + Dictyoptera), while sample_2_ represents (Zoraptera + Dictyoptera) group. We use μ to represent the population mean. Sample characteristics could be found in Table S1. Sample_1_ and Sample_2_ are both large samples (size above 100). According to the central limit theorem, these samples could be viewed as random variables which are normally distributed.

Table S1. Sample characteristics of Sample1 and Sample2

|  | Sample_1_ | Sample_2_ |
| --- | --- | --- |
| Size(n) | 304 | 414 |
| Mean(͞y) | 16.398 | 10 |
| Sample variance(s^2^) | 0.240 | 0 |

Set up the hypothesis:

H_0_: μ_1_=μ_2_

H_A_: μ_1_>μ_2_

Compute the u test statistic:


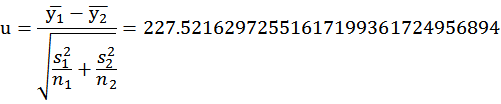


### Set up the rejection region of H_0_:

### As H_A_: μ_1_>μ_2_, use the upper tailed test. H_0_ is rejected when u>u_0.05_. According to table of Standard Normal Distribution Upper Critical Values, u_0.05_=1.645, u_0.005_=2.576.

### In this study, u>u_0.005_>u_0.05_, which means P<0.005, H_0_ is rejected. So the difference of the number of bases in the length variable region D3-4 within Polyneoptera, which distinguish between (Zoraptera + Dictyoptera) and the remain orders of Polyneoptera, is extremely significant.

**References**:

1. Schnare MN, Damberger SH, Gray MW, Gutell RR (1996) Comprehensive Comparison of Structural Characteristics in Eukaryotic Cytoplasmic Large Subunit (23 S-like) Ribosomal RNA. J Mol Evol 256: 701-719.
2. Taylor DJ, Devkota B, Huang AD, Topf M, Narayanan E, et al. (2009) Comprehensive molecular structure of the eukaryotic ribosome. Structure 17: 1591-1604.
3. Barker SC, Whiting M, Johnson KP, Murrell A (2003) Phylogeny of the lice (Insecta, Phthiraptera) inferred from small subunit rRNA. Zool Scr 32: 407-414.
4. Whiting MF, Carpenter JC, Wheeler QD, Wheeler WC (1997) The Strepsiptera problem: phylogeny of the holometabolous insect orders inferred from 18S and 28S ribosomal DNA sequences and morphology. Syst Biol 46: 1-68.
5. Johnson KP, Yoshizawa K, Smith VS (2004) Multiple origins of parasitism in lice. Proc R Soc Lond B 271: 1771-1776.
